# P-172. Assessing Dengue Vaccine Acceptance in Pediatric Caregivers in Kandy, Sri Lanka

**DOI:** 10.1093/ofid/ofaf695.396

**Published:** 2026-01-11

**Authors:** Caitlin Lawrence, Meghan Martin, Chamara Dalugama, Samidi Navaratna, Achintha Wanigasekara, Kevin Dieckhaus

**Affiliations:** University of Connecticut Medical School, Killingworth, CT; University of Connecticut Medical School, Killingworth, CT; University of Peradeniya, Peradeniya, Central Province, Sri Lanka; University of Peradeniya, Peradeniya, Central Province, Sri Lanka; University of Peradeniya, Peradeniya, Central Province, Sri Lanka; UConn Health, Southington, Connecticut

## Abstract

**Background:**

Sri Lanka experiences 60,000–100,000 cases per year, with high morbidity and financial burden. Despite the introduction of the DENGVAXIA® vaccine in the United States, vaccine hesitancy persists in certain regions due to safety concerns for seronegative individuals. A dengue vaccine has not yet been made available in Sri Lanka, and no prior studies have explored dengue vaccine acceptance in Sri Lanka. This study assessed caregiver knowledge and attitudes toward dengue vaccination in hospitalized pediatric patients, identifying factors influencing vaccine acceptance.

**Methods:**

A cross-sectional survey of 340 caregivers of hospitalized children (ages 0–16) was conducted in Kandy, Sri Lanka, during the dengue high season (June–August). Participants completed self-administered questionnaires available in English, Sinhala, and Tamil, with translators and researchers present. The survey assessed demographics, knowledge, attitudes, and practices regarding dengue and vaccination. Ethical approval was obtained from the University of Peradeniya and UConn Health. Statistical analyses included chi-square tests to identify relationships between demographic variables and vaccine attitudes.

**Results:**

Caregivers with higher educational attainment were significantly more likely to express willingness to vaccinate their child than those with lower education levels (p = 0.0024). Trust in physicians (p = 0.001), fear of dengue (p = 0.001), and positive general vaccine attitudes (p < 0.001) were also associated with higher willingness to vaccinate. Despite 50% of participants being unaware of a dengue vaccine, 57% expressed willingness to vaccinate if available, expressing high rates of physician trust (83%). No significant relationships were found between caregiver age, child age, or income and willingness to vaccinate.

**Conclusion:**

Caregivers’ willingness to vaccinate their children against dengue was influenced by physician trust, education level, and general vaccine attitudes. Findings highlight the importance of physician-led education campaigns to address vaccine hesitancy and promote vaccine safety. Future research should involve a larger, more diverse cohort and explore attitudes of caregivers who remain neutral toward vaccination.

**Disclosures:**

All Authors: No reported disclosures

